# Single-feature polymorphism discovery by computing probe affinity shape powers

**DOI:** 10.1186/1471-2156-10-48

**Published:** 2009-08-26

**Authors:** Wayne Wenzhong Xu, Seungho Cho, S Samuel Yang, Yung-Tsi Bolon, Hatice Bilgic, Haiyan Jia, Yanwen Xiong, Gary J Muehlbauer

**Affiliations:** 1 Supercomputing Institute for Advanced Computational Research, University of Minnesota, Minnesota, MN 55455, USA; 2 Department of Agronomy and Plant Genetics, University of Minnesota, St. Paul, MN 55108, USA; 3 USDA-Agricultural Research Service, Plant Science Research Unit, St. Paul, MN 55108, USA; 4 BASF Plant Science, Research Triangle Park, NC 27709, USA

## Abstract

**Background:**

Single-feature polymorphism (SFP) discovery is a rapid and cost-effective approach to identify DNA polymorphisms. However, high false positive rates and/or low sensitivity are prevalent in previously described SFP detection methods. This work presents a new computing method for SFP discovery.

**Results:**

The probe affinity differences and affinity shape powers formed by the neighboring probes in each probe set were computed into SFP weight scores. This method was validated by known sequence information and was comprehensively compared with previously-reported methods using the same datasets. A web application using this algorithm has been implemented for SFP detection. Using this method, we identified 364 SFPs in a barley near-isogenic line pair carrying either the wild type or the mutant *uniculm2 *(*cul2*) allele. Most of the SFP polymorphisms were identified on chromosome 6H in the vicinity of the *Cul2 *locus.

**Conclusion:**

This SFP discovery method exhibits better performance in specificity and sensitivity over previously-reported methods. It can be used for other organisms for which GeneChip technology is available. The web-based tool will facilitate SFP discovery. The 364 SFPs discovered in a barley near-isogenic line pair provide a set of genetic markers for fine mapping and future map-based cloning of the *Cul2 *locus.

## Background

Polymorphisms in DNA sequence between genotypes can be used as genetic markers for a variety of genetic studies. Directly sequencing genomes is one method of detecting these polymorphisms. For example, dideoxy sequencing resulted in the single nucleotide polymorphism (SNP) detections in human, mouse, and Arabidopsis [1-3]. High-throughput next-generation DNA sequencing technologies reduced the cost of and increased the efficiency of polymorphism detection [4-8]. High-density oligonucleotide resequencing arrays provide an alternative approach for polymorphism detection [9-11]. For example, the resequencing Genome-Wide Human SNP Array 6.0 [12] contains 906,600 potential SNPs that can be used to detect polymorphisms in individuals. However, this oligonucleotide resequencing array can only be developed for those species, such as human, mouse, Arabidopsis, and rice (*O. sativa*), whose genome sequences and the SNP map information are known [9-13]. Because of technology gaps and cost there is a lack of highly-parallel, high-throughput platforms for directly detecting DNA polymorphisms in many other species.

A polymorphic sequence detected by a single probe on an oligonucleotide array is called a single-feature polymorphism (SFP) [14]. With the emergence of microarray expression data, both gene transcript accumulation and SFP detection can be conducted at the same time. SFP discovery using microarray expression data is a rapid and cost-effective method for genetic marker development. The Affymetrix^® ^Corporation has developed more than 100 different types of commercially-available GeneChip expression arrays from over 30 organisms and additional customer-designed arrays [12]. Typically, Affymetrix^® ^GeneChips are designed with 11 perfect match (PM) and 11 mismatch (MM) probes (25-mers each probe) for each gene (probe set). Polymorphic nucleotides in the target transcript affect its binding to the probes, resulting in low hybridization signal intensity. Therefore, it is possible to identify SFPs between two genotypes by comparing these probe targeting polymorphic regions. By the same principle, genomic DNAs can also be applied to the oligonucleotide microarray for polymorphism detection [15,16].

Several methods have been reported for SFP discovery in a variety of organisms. All methods are based on the idea that variation in a target sequence lowers the probe hybridization signal intensity on an array. However, there are differences in methodologies to detect this decreased intensity. Winzeler *et al*. [15] first reported SFP detection in yeast (*Saccharomyces *cerevisiae) genomic DNA hybridized on a high-density oligonucleotide expression array. This method used a linear regression model to fit probe log(PM) intensities and then an F test to detect the probes that have significant differences in intensities between two yeast strains. This method has been tested on genomic and transcriptome data from *Plasmodium falciparum *and barley (*Hordeum vulgare *L.), respectively [16-18]. Borevitz *et al*. [14] developed a method that is similar to Significance Analysis of Microarray (SAM) [19] to analyze those probes that have significant differences in intensity between genotypes and detected SFPs in *Arabidopsis thaliana *genomic DNAs hybridized on a GeneChip. Coram *et al*. [20] recently applied a similar approach in wheat (*Triticum aestivum*) using the SAM method in the Bioconductor siggenes package [21] but detected the differences in probe intensities subtracted by the RMA (robust multichip analysis) [22] normalized-expression index. Rostoks *et al*. [23] modified this method for SFP discovery in barley by fitting PM intensities to a linear model (PILM) and then detecting the significantly-different probes using SAM in the Bioconductor package siggenes [21]. This method has been used for SFP discovery in mosquito (*Anopheles gambiae*) [24] and rice (*Oryza sativa*) [25], mapping qualitative and quantitative traits in *Arabidopsis thaliana *[26-31], and estimating mutation and recombination parameters in *Arabidopsis *[32]. Bischoff *et al*. [33] recently adapted this linear model by using SAS/JMP (SAS Institute Inc. Cary, NC) instead of SAM for SFP discovery in the swine transcriptome. Ronald *et al*. [34] implemented a positional-dependent-nearest-neighboring model (PDNN [35]) to calculate probe intensity and then examined the intensity differences of single pairs of probes for SFP discovery in yeast. All of these above methods are based upon changes in the probe signal intensity of individual probes between two genotypes.

West *et al*. [36] proposed a new method to detect SFPs in *Arabidopsis thaliana *by examining probe intensity along all 11 probes of a probe set in parallel and calculating a value called SFPdev, which is the hybridization signal difference between one probe and the average of the other 10 probes divided by the individual probe signal. Cui *et al*. [37] developed a probe affinity outlier pursuit (PAOP) algorithm by applying probe affinity differences and comparing 11 probes of a probe set to find the outliers for barley SFP detection. This method accounts for multiple probes in parallel in the same probe sets and uses probe affinity instead of probe intensity, and has been used for SFP discovery in cowpea (*Vigna unguiculata *L. *walp*) [38], mapping of translocation breakpoints in wheat [39], and genotyping in barley [40]. It is well agreed that a typical SFP has a "peak" profile shape, and almost all reports have shown such shape plots. However, none of the above methods were designed to directly detect and capture such an SFP "peak" shape.

Despite the ability to identify SFPs, a high false positive rate and low sensitivity have been observed with the above-mentioned SFP detection methods. For example, a 40% false discovery rate was estimated using the PILM method [23], and only "present" call probe sets were used in the PAOP method [37], suggesting a low sensitivity level. The objectives of our study were to: 1) develop and test a new SFP detection method that improves both the specificity and sensitivity; and 2) perform SFP discovery in a barley near-isogenic line (NIL) pair using the newly-developed method. We developed and tested our method on two previously published GeneChip datasets, including a dataset derived from six tissue types from the barley cultivars Golden Promise and Morex and a dataset of five genotypes (Barke, Morex, Steptoe, Oregon Wolf Barley Dominant and Recessive). We also used this method to detect SFPs in a barley NIL pair carrying the mutant and wild type alleles at the *Uniculm2 *(*Cul2*) locus.

## Results and Discussion

### Method development

Our SFP detection method, called the probe affinity shape power (PASP) method, is composed of two steps (source code is available by request: wxu@msi.umn.edu). We first detected probe affinity differences between genotypes over all probes on the GeneChip based on the model [37,41-43]:



where *S*_*tij *_is the raw PM intensity, *I*_*ti *_is the RMA-normalized [22] expression value of each gene, and *t *represents the genotype, *i *represents probe set, and *j *the probe. The error factor *E *is an independent identically-distributed error with a mean of zero, and the estimation of probe affinity value (*A*) can be calculated by subtracting *I *from *S*.

We used the Bioconductor affy package [21] for PM intensity extraction and RMA-normalized expression calculation [22]. All PM intensities were background subtracted and quantile-normalized [44]. Each probe feature affinity was computed by subtracting the normalized probe intensity by the RMA expression value of that probe set. A probe affinity matrix was created with sample numbers in columns and probe sets multiplying by 11 in rows. The SAM method in the Bioconductor siggenes package [21] was applied to detect significant differences of each probe affinity among all samples in the probe affinity matrix. The estimated prior probability (*p*_0_) that a feature does not bear significant difference in probe affinity was set to 0.95. Using the Barley GeneChip, we detect 250,811 probes at the same time in the SAM method, an FDR is deduced by applying multiple testing [19,45] to the raw *p *value of each probe. We used the FDR instead of the significant p value as the cutoff. The FDR was set to 0.1 based on the delta value (*D*) in the SAM analysis. The FDR cutoff values are arbitrary, and 0.1 is commonly acceptable. More stringent cutoff values can be applied.

The second step of PASP was to capture the probe affinity profiles (shape powers) around the probe that produced significant affinity difference, and to generate a SFP weight score. For each probe that showed significant difference in affinity with an FDR cutoff of 0.1 in SAM, the probe affinities at adjacent positions in the same probe set of all samples were extracted. A two-value direction vector *V *was created for each genotype, *V*_1 _and *V*_2_.



where *L *is the probe affinity in the left position, and *R *is the probe affinity in the right position of the potential SFP.

In each genotype, if the median of all probe affinity values in the adjacent location (*md*(*A*_*i*-1_) or *md*(*A*_*i*+1_)) is higher than the median affinity of the potential SFP location *i*, a value of 1.0 was assigned to the appropriate index (*L*_1_, *L*_2 _or *R*_1_, *R*_2_) of the vector. If the probe median affinity of the adjacent location is lower than those at the SFP location, a value of negative one was assigned to the vector. We tested the minimum values of 0.01, 0.05, 0.1, and 0.2 in determining the affinity difference, and found these values did not exhibit significant difference in the performance of SFP detection (Additional file 1). We chose the empirical value of 0.1 as a more reliable minimum value. If the affinity difference is less than or equals 0.1, or if the adjacent location is over the maximum boundary when a potential SFP is at probe position 1 or 11, the adjacent probe median affinity was set to 0 in the vector.





To take into account the direction variation of probe affinities in the median calculation, we computed the proportion (*p*) of all (*n*) individual probe affinity directions that have the same direction to the median affinity direction, and multiplied the median direction vector with this proportion.

The shape power *P *is the sum of the absolute values of the two vector subtraction.



The minimum value of 1.0 is assigned to the shape power to ensure the original weight at least when a probe does not exhibit a typical affinity shape. The original weight is the SFP affinity difference (*Ad*) divided by the 30^th ^percentile (30 *pct*) of affinity differences of the 11 probes within the same probe set. We set a minimum value of 0.1 to the 30^th ^percentile to avoid an extreme large weight score because of an extreme small 30^th ^percentile division. The 30^th ^percentile was optimized to ensure possible multiple SFPs per probe set even though the 30^th ^percentile base line only shows a little bit better performance than other different percentiles at a point of weight score cutoff of 2.5 (Additional file 2). Within the 11 probe affinity values of each probe set, the 30^th ^percentile is the 4^th ^lowest probe. Using the 4^th ^lowest probe as the base allows capture of the remaining 7 probes on the same probe set. We found if more than 8 probes were detected on the same probe set, they were most likely caused by differential expression instead of sequence variation (data not shown).

The final SFP weight score *W *is this weight multiplied by the shape power (*P*) to the power of two.



Specificity and sensitivity are two important criteria in any detection method development. In the SFP discovery methods reported to date, low specificity and low sensitivity are the common problems. Even though PASP is closest to PAOP, the PAOP method has only been shown to function for probe sets with present calls [37]. In general, the Barley1 GeneChip exhibits approximately 60% present calls. Some platforms have a low percentage of present call probe sets. For example, the Wheat GeneChip typically has about 45% present calls, while the Medicago GeneChip has approximately 40% present calls (data not shown). If a platform is used for cross species hybridization, the present calls will be even lower [38]. Therefore, if only present calls are taken into account a lower sensitivity would result and many SFPs would escape detection. The "present/absent" call does not necessarily mean present/absent in terms of hybridization but an Affymetrix definition that a probe set is called absent if there is no significant difference with a *p *value cutoff of 0.04 between the 11 PM probes and 11 MM probes [46]. In our method design, we first used probe affinity difference instead of probe signal intensity to increase the sensitivity, and then applied shape power scores to increase the specificity. In cases where both PM and MM show no significant difference but both have high intensities, PASP is still capable of detecting SFPs even with an absent call probe set, thus the sensitivity was enhanced. In the case of differentially-expressed genes, after subtracting the expression index (RMA), all the probe affinities are comparable between the two genotypes. If SFPs existed, their probe affinities would be captured. However, these SFPs might escape if only the probe intensities are detected or only present call probe sets are used.

The motivation of the affinity shape power is the following. The SFP probe has different affinity to different genotype targets, and this affinity can exhibit a sharp contrast in neighboring probes within the probe set that are not SFPs. If all sample replicates present this sharp contrast at this probe, this potential SFP bears more power in weight score. In cases that most or all probes within the probe set have different signal intensities between genotypes but do not form affinity contrast with neighboring non-SFP probes, these potential SFPs bear less or no power in the weight score since they might be caused by differential transcript accumulation. Thus, the probe affinity shape power captures the intrinsic feature of SFPs.

When using genomic DNAs instead of transcripts to hybridize on high density GeneChip arrays, the probe intensities can be also summarized to an index by RMA, thereby the affinity shape powers can be computed by our method.

### Method validation

To validate our method, we tested previously-published Barley1 GeneChip expression data sets [23]. Thirty-six GeneChip hybridizations (cel files) from two genotypes, Morex and Golden Promise, were downloaded from the public barley Natural variation web site [47]. For each genotype, six tissue types (coleoptile, crown, embryo, leaf, radicle, and root) were examined. Three biological replicates were examined for each genotype/tissue combination except for root tissue which has two replicates of Morex and four replicates of Golden Promise. This data set was accompanied by 401 polymorphic and 2200 non-polymorphic probes previously sequence-verified between Golden Promise and Morex.

With PASP method, a total of 9,603 SFPs (Additional file 3) were identified between Golden Promise and Morex. To determine the detection sensitivity and specificity, we examined Golden Promise and Morex at known polymorphic- and non-polymorphic-sequences. We combined all data from the six tissues. As seen in Table 1, there are a total of 250,811 probes on the Barley1 GeneChip, and after the first step of probe affinity difference detection by SAM with a 10% FDR cutoff, of the 401 known polymorphisms, 305 (76.06%) were detected, while only 84 of the 2200 non-polymorphic sequences were detected as false positives.

**Table 1:** Single-feature polymorphisms (SFPs) discovered in Golden Promise and Morex^1^

	**Probes**	**Polymorphic**	**Non-polymorphic**
	
GeneChip^2^	250,811	401	2,200
SAM FDR0.1^3^	15,026	305	84
Weight score2.5^4^	9,603	284	48
Rate (%)^5^	3.82	70.82	2.18

^1 ^Barley1 GeneChip transcript data obtained from two genotypes (Morex and Golden Promise) from six tissue types [47] were used in this analysis.

^2 ^GeneChip indicates the Barley1 GeneChip which contains 250,811 probes. There are 401 polymorphic probes and 2200 non-polymorphic probes sequence verified between Morex and Golden Promise in this dataset [47].

^3 ^SAM FDR0.1 indicates the number of SFPs detected using a false discovery rate (FDR) 0.1 in the SAM (significance analysis of microarray) analysis of the bioconductor siggenes package.

^4 ^Weight score 2.5 indicates the number of SFPs detected after a weight score cutoff value of 2.5 was applied.

^5 ^Rate (%) indicates the percent SFPs with a weight score cutoff of 2.5 divided by the number of polymorphic, non-polymorphic, or all probes.

**Figure 1:**
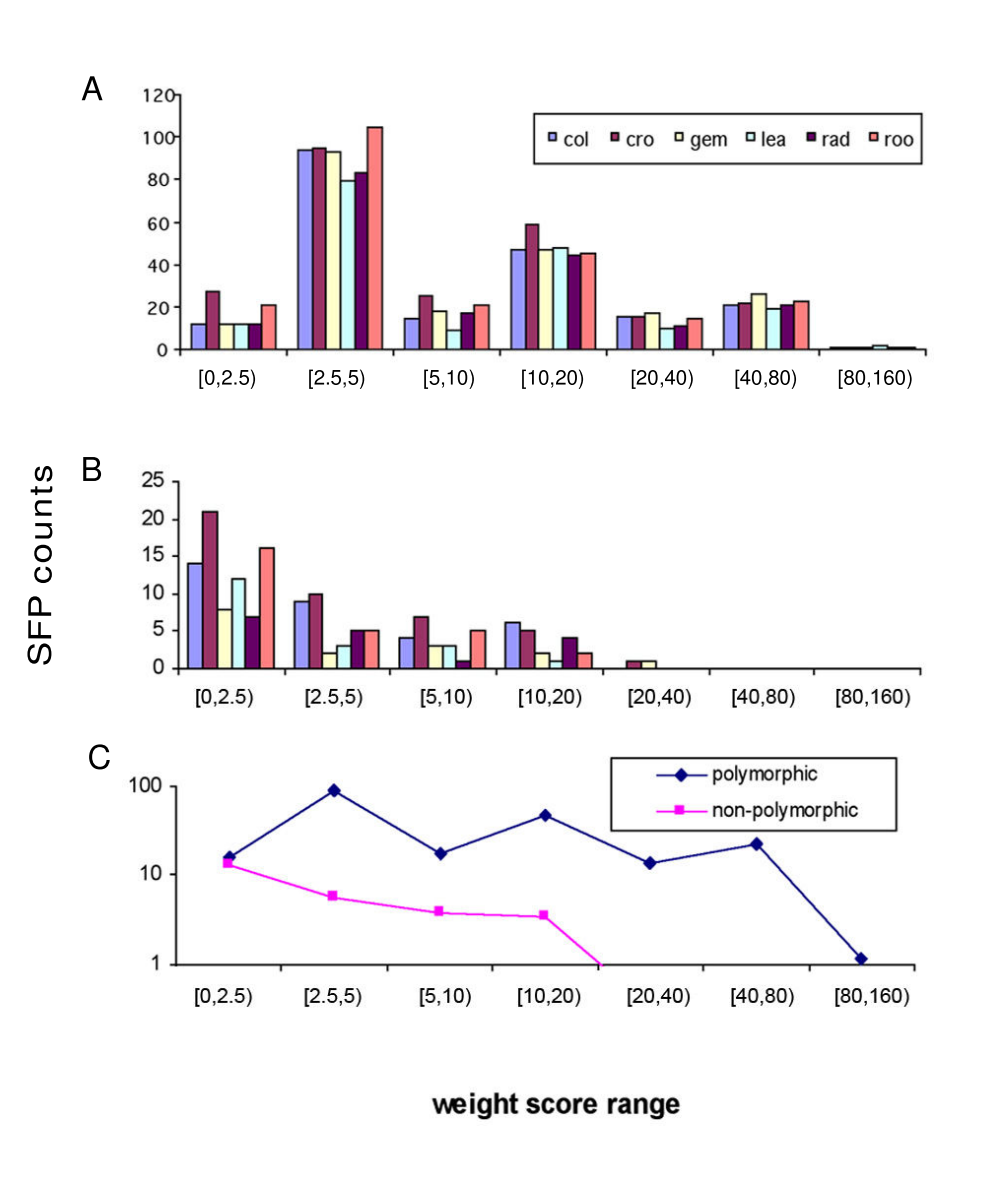
**Weight score distribution of known polymorphic and non-polymorphic probes**. The weight score distribution of the 305 known polymorphic probes (A) and 84 known non-polymorphic probes (B) contained in the potential SFP list that passed the cutoff false discovery rate (FDR) of 0.1 in the SAM (significance analysis of microarray) analysis. The curves with averaged counts of sequence confirmed polymorphic probes (true SFPs) and sequence confirmed non-polymorphic probes were shown (C). The probe numbers were plotted in different weight score bin intervals. Tissues: col, coleoptile; cro, seedling crown; gem, embryo from germinating seed; lea, seedling leaf; rad, radicle; roo, seedling root.

The probe affinity shape power score contributed to the improved specificity and sensitivity of PASP method. As seen in Figure 1A, most of the 305 known polymorphic probes (true positives) contained in the potential SFP list from the SAM analysis had weight scores above 2.5 in each tissue. Only a small number of these probes were below a score of 2.5. The 84 non-polymorphic probes (false positives) contained in this potential SFP list exhibited an opposite trend as most have scores below 2.5 (Figure 1B). The curve of averaged counts of true positives (polymorphic probes) and the curve of averaged counts of false positives (non-polymorphic probes) converged at a score below 2.5 (Figure 1C). Based on this observation, we applied a weight score of 2.5 as the cutoff in the discovery of individual tissue SFPs. This cutoff optimized a high sensitivity and low false positive rate at the same time.

**Figure 2:**
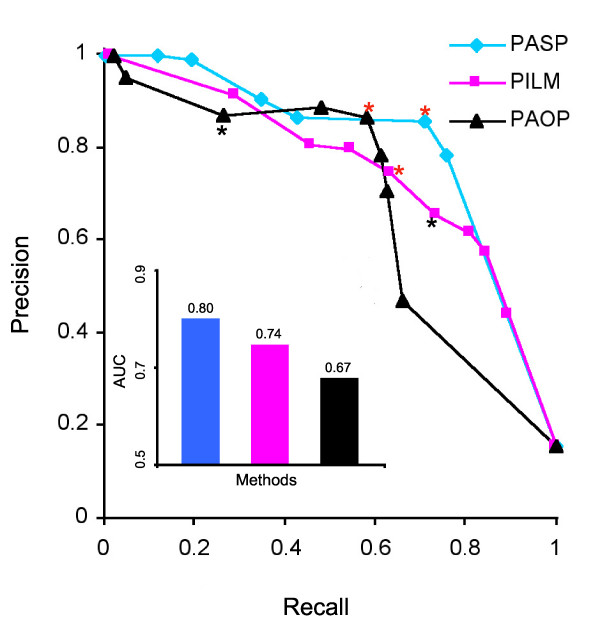
**Detection accuracy performances**. Precision-Recall (PR) curve shows precision levels (y-axis) at different Recalls (or true positive rate TPR, x-axis). PASP line (blue), PILM (red), and PAOP (black) were plotted by 8, 10, and 9 selected weight score cutoffs (Additional file 4). The points with the best performances are indicated by red stars and the cutoff values reported in original methods are indicated by black stars. The Areas Under the Curve (AUC) were indicated by the bar chart.

We calculated the Precision-Recall (PR) curve [48] to further examine the performances at different weight score cutoffs. As seen in Figure 2, curves tending to the upper-right-hand corner exhibit better performance. The 2.5 cutoff point on the PASP line (Figure 2, blue line, star) shows the highest performance compared to all other points on the PASP line.

After imposing a weight score cutoff of 2.5, the number of false positives decreased from 84 to 48, with only a loss from 305 to 284 true positive SFPs (Table 1). As a result, a total of 284 SFPs were detected from 401 known polymorphic probes, resulting in a sensitivity rate of 70.82%. A total of 48 false SFPs were called from 2,200 confirmed non-polymorphic probes for a false positive rate (FPR) of 2.18%. The FDR is 14.46%, which was calculated by the number of false positives divided by the sum of false positives and true positives, 48/(48+284).

**Figure 3:**
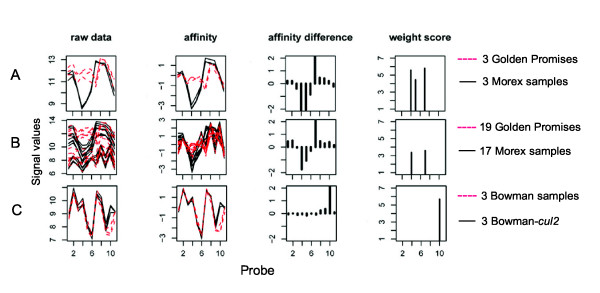
**Single-feature polymorphism (SFP) plots**. A: Probe set Contig11534_at in three Golden Promise samples (broken red) compared to three Morex samples (solid black). B: Contig11534_at in 19 Golden Promise samples (broken red) compared to 17 Morex samples (solid black) from six different tissue types. C: Contig16609_at in three barley Bowman samples (broken red) compared to three Bowman-*cul2 *samples (solid black). The x-axis represents the 11 PM probes. The y-axis represents log_2 _intensity of perfect match (PM) probes before normalization (raw data panel), the log_2 _normalized intensity subtracted by the expression index of that probe set (affinity panel), the probe affinity (affinity difference panel), the weight score of the SFPs after the 2.5 cutoff (weight score panel). The three SFPs detected in Contig11534_at (4,5,7) contain verified polymorphisms between barley Golden Promise and Morex (A, B). The SFP Contig16609_at probe 10 in barley Bowman-*cul2 *was verified by sequencing (C).

PASP can also detect multiple SFPs in the same probe set even though more than 50% of the SFPs discovered were single SFPs in one probe set. For example, one and two SFPs in Contig11534_at were detected in Morex and Golden Promise, respectively (Figure 3A). These three SFPs were among the 401 sequence-verified polymorphic probes. However, when we pooled all 17 versus 19 samples of the six different tissues together, one of the SFPs in Contig11534_at (probe 5) could not be detected (Figure 3B). Because there are variations at probe-level among different tissues in the same genotype, these variations within genotype or standard deviation (SD) caused by pooling multiple tissue samples will diminish the statistical difference between genotypes. This observation is consistent with previous observations that the discovery of SFPs in individual tissues may reduce the probe-level variation in expression across tissues [23], hence it is more sensitive than pooling multiple tissue samples together. Some gene loci that are expressed at low levels in one tissue or stage may be transcribed at high levels in other tissues or stages. Thus, using multiple tissue or stage samples for separate testing can increase SFP detection.

### SFP discovery method comparison

To date, there are several SFP discovery methods reported. Among them, the PILM methods developed by Rostoks *et al*. [23] and the PAOP method developed by Cui *et al*. [37] have been repeatedly used [14,20,23-33,37-40]. We compared the PASP method developed in this study to these two SFP discovery methods by using the GeneChip dataset of two barley genotypes Morex and Golden Promise, originally used by Rostoks *et al*. [23,47].

We plotted the PR curve to compare the detection accuracy performances. The PR curve (Figure 2) shows the PASP method line reached the upper-right-hand corner, and there is not much room for further improvement in SFP detection. The PAOP and PILM are under the PASP line. The areas under the PR curves from PASP, PILM, and PAOP are 0.80, 0.74, and 0.67, respectively (Figure 2, Additional file 4). These areas under the curve (AUC) values represent the overall performances of these three methods. We also tested the Receiver Operator Characteristic (ROC) curve to compare the detection accuracy performances of these three methods (data not shown). While the PR and the ROC are equivalent, the PR curve is more informative when dealing with highly skewed datasets [48]. Thus, we only showed the PR curve.

We further compared PASP with PAOP and PILM by using the cutoff values that produce the best performance for each method. PILM exhibits its best performance at cutoff value of delta (*D*) 3.0 (red star on PILM line). We chose both the *D *cutoff of 3.0 (FDR 0%) and *D*2.0 (FDR 0.1%, black star) for the PILM comparisons since *D*2.0 was optimized in the original report [23]. PAOP shows its best sensitivity and specificity at outlier score percentile of 0.15 (os0.15, red star) even though os0.05 (black star) was used in the original report [37].

As shown in Table 2, we found a high FDR of 34.46% using the PILM method (cutoff *D*2.0), which is close to the FDR estimation (~40%) in the original report [23]. At its best performance cutoff (*D*3.0), the FDR is still high (25.51%). The PAOP showed a lower FDR either at the cutoff of os0.05 (FDR 13.11%) that was optimized in the original report [37] or at the cutoff of os0.15 (FDR 13.70%) that produced its best performance. However, its sensitivity (TPR) and detected SFP number are relatively low in either case. However, the PASP has a low FDR (14.46%) at a sensitivity level of approximately 70% when the same dataset was tested (Table 2).

**Table 2:** Single-feature polymorphism (SFP) discovery methods comparison

	Methods				
	
	PASP	PILM	PILM	PAOP	PAOP
	probe affinity, shape power	probe intensity, linear model	probe intensity, linear model	probe affinity, outlier score	probe affinity, outlier score
cutoff	weight2.5	D3.0 FDR0%^2^	D2.0 FDR0.1%^1^	os pct0.15^2^	os pct0.05^1^
polymorphisms	284/401	254/401	295/401	233/401	106/401
non-polymorphisms	48/2200	87/2200	155/2200	37/2200	16/2200
detected SFPs	9603	7150	10504	6820	2193
TPR (%)	70.82	63.34	73.56	58.10	26.43
FPR (%)	2.18	3.95	7.05	1.68	0.73
FDR (%)	14.46	25.51	34.44	13.70	13.11
compScore	1.00	0.21	0.15	0.80	0.28

The PASP (probe affinity shape power) method developed in this study was compared with the PILM (probe intensity linear model) and the PAOP (probe affinity outlier pursuit) methods using the same dataset used in the previous study [23,47]. Both the cutoff values used in original report ^1 ^and the cutoffs at which the best performance was produced ^2 ^in each method were tested. The 401 polymorphic and 2200 non-polymorphic sequences of Golden Promise and Morex were derived from the previously-published Barley1 GeneChip data sets [47]. The calculations for true positive rate (TPR), false positive rate (FPR), false discovery rate (FDR), detected SFPs, and compScore are described in the Materials and Methods section. CompScore is the comparison of the PASP method versus each of the other methods.

We computed a comparison score (compScore, see Materials and Methods) for these methods by combining the sensitivity, specificity, FDR, and the discovered SFP number that are calculated using the cutoff values at which the best performance was achieved in each method. The comparison score shows a percentage of improvement or reduction in performance compared to other methods. A score of 1.0 means that the two methods have the same performance. A score of less than 1.0 reflects reduced performance overall. As seen in Table 2, the compScore of the PASP method is better over all methods tested. We adjusted the original cutoff value of the PAOP method from 0.05 to 0.15 to compensate for the sensitivity by sacrificing its original high specificity. At this cutoff, even though the PAOP method shows a little bit better specificity (lower FDR and FPR) than PASP, it has lower sensitivity and less discovered SFPs than PASP. The compScore is less than 1.0 (0.80, Table 2), therefore the overall performance of PAOP is lower than PASP. Since the PILM method had a high FDR, it produces a very low compScore (0.21) even at its best performance cutoff (*D*3.0), *i.e*., the overall performance of PILM in terms of specificity, sensitivity, and detected SFPs is only 21% of that of PASP. The overall compScore computed from the best point for each method shows that PASP is superior to the other two methods (Figure 2).

**Figure 4:**
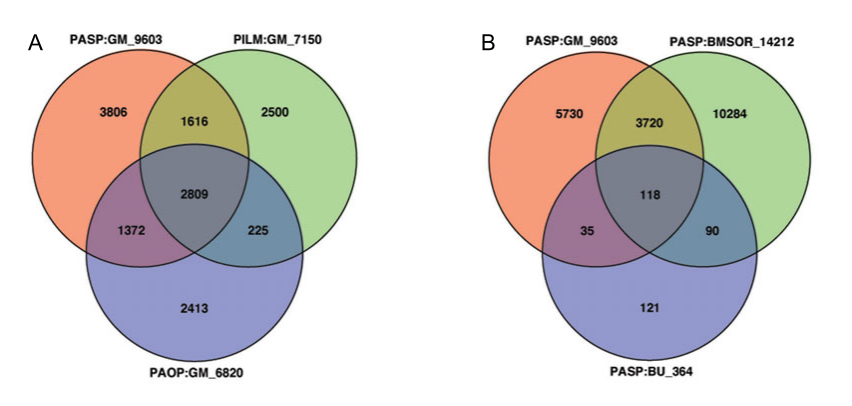
**Comparison of different single-feature polymorphism (SFP) detection methods**. A: 9603 SFPs detected in Golden Promise and Morex by probe affinity shape power (PASP) method compared with 7150 SFPs detected by probe intensity linear model (PILM) method, and 6820 SFPs by probe affinity outlier pursuit (PAOP) method. The best performance cutoff values were used for each method. B: Comparison of 364 SFPs discovered by PASP in Bowman and Bowman-*cul2 *with 9603 SFPs discovered in Golden Promise and Morex, and 14212 SFPs discovered by PASP in Barke, Morex, Steptoe, and Oregon Wolf Barley Dominant and Recessive.

The weight score cutoff of 2.5 on the PASP line (red star) of PR curve exhibits superior performance to any points on the PILM or PAOP lines (Figure 2). The 9603 SFPs discovered by PASP at this cutoff represented robust SFPs, which are more than the SFPs discovered by PILM (7150) and by PAOP (6820) at their best performance cutoff values. Approximately one-third (2809) of the SFPs were found overlapped using the three methods, and another one-third of the SFPs were detected by a combination of any two methods (Figure 4A). More than one-third of the SFPs (3806) discovered by PASP were not detected by either PILM or PAOP.

We also tested our method on the same dataset published by Cui *et al*. [37] using the same genotype pair comparison. In the previous study, a total of 2,007 SFPs were reported using a highly stringent cutoff value (5 percentile) [37]. Using our PASP method we detected significantly more SFPs (14,212). These SFPs covered 96.6% (1939 out of the 2007) of the SFPs discovered by the PAOP method [37]. This would seem to call into question the number of false positives in the remaining 12,273 SFPs identified using the PASP method. However, when we compared SFPs between Morex and Golden Promise, we showed that our PASP method using a weight score cutoff of 2.5 has a much higher sensitivity (70.82%) than PAOP (26.43%) using a 5 percentile cutoff at a similar FDR (14.46% versus 13.15%) (Additional file 4, Figure 2). In addition, the Cui *et al*. [37] study pointed out that the five different genotypes (Barke, Morex, Steptoe, Oregon Wolf Barley OWB Dominant and OWB Recessive) exhibit a high level of polymorphisms and it is possible that not all SFPs were detected. Thus, the PASP method captures more SFPs in addition to almost all of the previously-identified SFPs using the PAOP method. Again, there could be more false positives in the 14,212 SFPs detected using the PASP method than in the 2,007 SFPs using the PAOP method. However, the PASP method provides an additional set of SFPs to examine.

Since cRNA-based SFP detection is based on the probe hybridization signals, 100% sensitivity can never be reached because there are many genes that exhibit low transcript accumulation. The maximum sensitivity in SFP discovery is determined by the percentage of genes (probe sets) on the array that exhibit signal. The 70% sensitivity that we obtained may be the maximum in barley while maintaining a low FDR (Figure 2, Table 2).

We compared the PASP method with two commonly used methods because these methods have been extensively used in various studies [14,20,23-33,37-40]. Other methods including the PM-MM model method [17], the K-mean clustering method [17], the method developed by Winzeler *et al*. [15], and the method by Ronald *et al*. [34] were compared in a study by Luo *et al*. [17] using barley data set. Each of these methods exhibited a high (~64%) FDR [17,33] and a low sensitivity (27%–37%). West *et al*. [36] described a SFPdev method by analyzing an *Arabidopsis *dataset, but these authors did not extensively test for FDR or sensitivity through sequence information. In contrast, the PASP method retained a high sensitivity but lowered the FDR at the same time, and therefore seems to provide a better performance over previous methods.

### Implementation of the Web-based SFP discovery tool

We implemented the PASP method as a public web application tool . The web application makes SFP detection easy for potential users. Job over-load on the server is a common issue in web application design, especially for high-throughput genomic analysis. Our SFP discovery tool design consists of a client, web server, and application server three-tier components. The jobs are scheduled in a queue on the application server by the web server to avoid the over load issue. The interface has an add-more button allowing for loading a flexible number of files. An email address text field is provided to inform the user of the URL link for downloading the result files when the job is done.

### Discovering SFPs in a near-isogenic line pair carrying mutant and wildtype alleles for *uniculm2*

Four tissue types (crown, embryos, immature inflorescence, and 3 day old seedlings) of the NIL pair carrying the wild type (Bowman) and mutant allele for *cul2 *(Bowman-*cul2*) were used in this study. RNA was extracted from these four tissues with three replicates and hybridized to the Barley1 GeneChip^® ^Genome Array.

GeneChip cel files from the hybridizations were quality ensured using GCOS v1.4 (Affymetrix, Inc. Santa Clara, CA 95051 USA) analysis. The R script first detects all probes with significantly different probe affinities by SAM FDR cutoff of 0.1, and then the SFP weight score 2.5 was applied to each of the putative SFPs. As seen in Table 3, SFPs were detected in both Bowman and Bowman-*cul2*. The SFP numbers in the two lines were very close, each accounting for approximately half of the total SFPs. SFPs detected in the four tissues were combined for a total of 364 SFPs (Table 3, Additional file 5). These SFPs were identified in 263 probe sets, 132 probe sets in Bowman-*cul2 *and 146 probe sets in Bowman, and some SFPs were located in probe sets from both.

**Table 3:** Single-feature polymorphisms (SFPs) discovered in Bowman and Bowman-*cul2*

	# of SFPs discovered in individual issues				
		
Genotype	crow	embr	infl	seed	Total
*Cul2*	76	112	104	79	183
Bowman	64	98	97	63	181
					
Total	140	210	201	142	364

The SFPs detected in four tissues that exhibited a weight score of 2.5 or greater (crow, crown; embr, embryo; infl, inflorescence; and seed, seedling).

More than half (187) of the SFPs were found as a single SFP in a probe set. An example of a single SFP in a probe set is shown in Figure 3C. This SFP (Contig16609_at probe10) was found in Bowman-*cul2 *and has an SFP shape with a weight score of 64 (Figure 3C). A maximum of six SFPs per probe set were detected (data not shown). In probe sets exhibiting multiple SFPs, some SFPs were present in one genotype only, and some probe sets possessed SFPs in both genotypes.

### Validation of the SFPs discovered in Bowman and Bowman-*cul2*

A subset of the SFPs identified in Bowman and Bowman-*cul2 *were sequence verified. Sixteen SFPs located in 13 probe sets were targeted. PCR primers were designed for these 13 probe sets with an amplicon size of 200 to 300 bp (Additional file 6). Of the 16 targeted SFPs, nine were in Bowman-*cul2 *and seven in Bowman. Fifteen out of the 16 SFPs were validated as true positives by the sequence data. The only one false positive SFP (Contig8825_at probe 7) showed a low weight score compared to other validated SFPs (Table 4).

**Table 4:** PCR sequence verification for 16 single-feature polymorphisms (SFPs)

SFPs^1^	Tissue^2^	Genotype^3^	Shape^4^	Weight^5^	PCR^6^	Map info^7^
Contig16609_at10	3	U	4	85.45	+	NA
Contig4329_at3	4	B	4	70.77	+	6H,L, 67.7 cM
Contig4329_at6	2	B	4	71.33	+	6H,L, 67.7 cM
Contig5339_s_at8	4	B	4	65.08	+	6H,L, 70 cM
Contig7178_s_at8	4	U	3.67	50.82	+	6H,L, 70 cM
Contig4107_x_at11	4	U	2	23.01	+	NA
Contig9366_at10	3	U	2.67	39.79	+	NA
Contig159_at1	4	U	2	15.03	+	NA
Contig159_at6	3	U	1	3.78	+	NA
Contig9298_at7	2	U	1	3.64	+	NA
Contig14687_at7	3	B	3	37.18	+	6H,L, 71.1 cM
HVSMEg0016A12r2_s_at5	4	B	4	75.24	+	6H,S, 49.4 cM
HVSMEg0016A12r2_s_at7	3	B	2	16.13	+	6H,S, 49.4 cM
HVSMEn0016F09r2_s_at7	1	U	1	4.48	+	6H,L, 71.1 cM
Contig2856_x_at6	3	U	1.67	8.16	+	6H,L, 60.2 cM
Contig8825_at7	3	B	1	5.48	-	3H,L, 55.6 cM

^1^Indicates the contig and probe that the SFP was detected.

^2^The tissue column indicates the number of tissues out of four where the SFP was discovered.

^3^Genotype represents the SFP detected in Bowman (B) or in Bowman-*cul2 *(U).

^4^The shape power and ^5^weight score were calculated as described in the Materials and Methods section.

^6^The SFP was verified (+) or not verified (-) by sequencing PCR products.

^7^SFP map information was derived from barley EST Assembly #35 at the HarvEST website [50]. Map locations on chromosome 6H or others were designated as long (L) or short (S) arm, at length cM, unless mapping information was not available (NA).

It is impractical to perform sequencing to validate every SFP call. To further determine the reliability of the SFPs discovered in the two genotypes, we examined other genotypes. We found that nearly one third (118) of the 364 SFPs we discovered between the Bowman and Bowman-*cul2 *genotypes exist in Golden Promise, Morex, Barke, Morex, Steptoe, or the Oregon Wolfe barleys (Figure 4B).

Since true SFPs possess a typical shape, we reviewed the probe affinity shape plots of all SFPs to justify each SFP. A quantitative likely number can also be assigned to each SFP discovered. West *et al*. [36] reported an SFPdev by calculating the probe signal rate after the subtraction from the average probe signal. Cui *et al*. [37] reported the probe outlier values. Statistic p value or FDR was also used to rank SFPs [17,23]. In this study, we present an SFP weight score. The weight score computed for each SFP represents how likely that the SFP is real, *i.e*., an alternative confirmation of SFPs. From the published barley expression data set, a weight score cutoff of 40 resulted in a 0% false positive rate. For SFP detection in each tissue type of Bowman and Bowman-*cul2*, between 30 to 45 SFPs possessed a weight score of 20 or above, resulting in a total of 89 SFPs with weight scores at 20 or above among all 364 detected SFPs. About half of the SFPs had a score of 10 or above.

### SFP-containing genes on chromosome 6H

**Figure 5:**
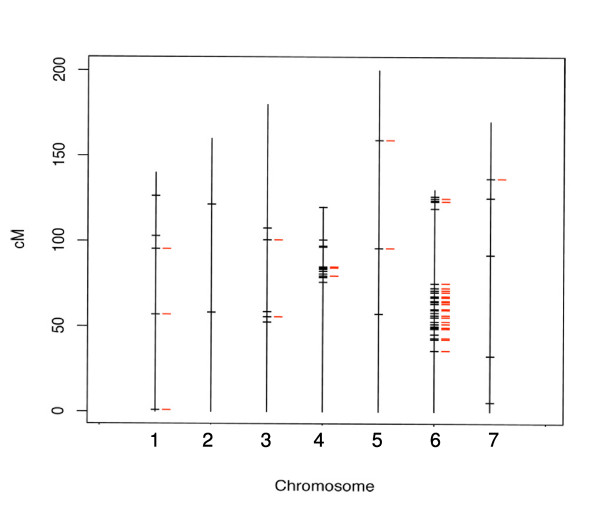
**Map location of SFPs detected between Bowman and Bowman-*cul2***. A total of 91 out of 263 single-feature polymorphism (SFP) probe sets discovered between Bowman and Bowman-*cul2 *mutant were mapped on barley chromosomes 1H to 7H using the map information from HarvEST: barley version 1.68 [50]. Black: Mapping of 91 SFP-containing genes in which SFPs have weight score cutoff of 2.5. Red: Mapping of 65 SFP-containing genes in which SFPs have weight score of 20.0 or greater.

Bowman-*cul2 *and Bowman are a NIL pair derived from five backcrosses of the *cul2 *mutant to Bowman allele. Thus, we would expect that a substantial number of SFPs would map in the introgressed region surrounding the *Cul2 *locus. *Cul2 *was previously mapped on chromosome 6H Bin 6 [49]. We searched the available mapping information for the Barley1 probe sets by using HarvEST:Barley software version 1.68 and barley EST Assembly #35 at the HarvEST website [50]. A total of 2,905 Contigs with 2,943 SNPs have been mapped on seven chromosomes [51]. Among the 263 SFP probe sets containing 364 SFPs discovered between Bowman-*cul2 *and Bowman, we found map information for probe sets that correspond to 91 probe sets (Additional file 7) containing 133 SFPs. Seventy-eight SFPs from 50 SFP probe sets were mapped to chromosome 6H. Even though we did not have map information for all 364 SFPs of the 263 SFP probe sets, this result clearly demonstrated that polymorphisms occurred more frequently on chromosome 6H in the vicinity of the *Cul2 *gene (Figure 5, Additional file 7). The fact that most of the SFPs were identified on chromosome 6H Bin 6 helps to validate the reliability of the SFPs.

There are two clusters of SFPs on chromosome 6H (Figure 5, black). Eight SFP-containing genes (10 SFPs) are clustered in a region of approximately 7 cM in length from 119 cM to 126.2 cM. The second cluster has 42 SFP-containing genes (68 SFPs) clustered in a region of approximately 39 cM in length from 35.8 cM to 75.2 cM. Among the 15 SFPs that were validated by PCR sequencing, nine SFPs are in this region. The nine SFPs are represented by seven genes (Contig4329_at 67.7 cM, Contig5339_s_at 70 cM, Contig7178_s_at 70 cM, Contig14687_at 71.1 cM, HVSMEg0016A12r2_s_at 49.4 cM, HVSMEn0016F09r2_s_at 71.1 cM, Contig2856_x_at 60.2 cM). Gene KFP128 (Contig2856_x_at) was found to be closely linked to *Cul2 *locus within 4.6 cM [49]. The remaining six sequence-validated SFPs do not have map information. Interestingly, the only SFP that was not confirmed through sequencing was mapped on chromosome 3H.

We also found SFPs on other chromosomes such as chromosome 4 (Figure 5). This reflects that the Bowman-*cul2 *may still carry progenitor regions of genome sequences even after five backcrosses of the *cul2 *mutant allele into Bowman. The SFPs found on the other chromosomes may be the result of genetic variation between Bowman and the progenitor sequences. Another possibility is that the SFPs with low weight scores may contain false positives. We tried a more stringent SFP weight score cutoff of 20.0 that resulted in 113 SFPs harbored in 65 probe sets that had mapping information. We plotted these 65 SFP-containing genes on to barley chromosomes and found only one polymorphic cluster with 33 SFP-containing genes representing 45 SFPs on chromosome 6H Bin 6 in the vicinity of *Cul2 *locus. The other chromosomes only have few SFPs (Figure 5, red).

Barley plants that carry loss-of-function mutations in the *Cul2 *gene result in plants that do not tiller (vegetative branches) [49]. The *Cul2 *gene has not been cloned yet and the genetic components of tiller development in barley are unknown. The 68 SFPs in 42 genes discovered in the region from 35.8 cM to 75.2 cM on chromosome 6H provide a potential marker list for future map-based cloning of the *Cul2 *locus.

## Conclusion

We developed a new robust method for SFP discovery and tested the method using Barley1 GeneChip datasets. This SFP discovery method can be used for other organisms for which GeneChip technology is available. Our result clearly showed our new SFP discovery method is superior to previously-reported methods. The web implementation of this method will provide a resource for others to employ the PALP algorithm for SFP detection. The 364 SFPs discovered in this study between plants carrying the wild type and mutant allele for the *Cul2 *gene provide a potential marker list for fine mapping and future map-based cloning of the *Cul2 *locus.

## Methods

### Genetic stocks

The barley cultivar Bowman was a gift from J. Franckowiak, Department of Plant Sciences, North Dakota State University, Fargo, N.D. The Bowman-*cul*2 genetic stock carries a single gene recessive mutation at the *Cul2 *locus. The *cul2 *mutation was backcrossed five times into the Bowman genetic background to create Bowman-*cul2*. The Bowman-*cul*2 stock (GSHO2038) was obtained from USDA-ARS, National Small Grain Germplasm Research Facility, Aberdeen, Idaho.

### Experimental design for detecting SFPs in Bowman and Bowman-*cul2*

Four tissues were collected at different stages for Bowman and Bowman-cul2: whole seedlings at 2–3 days after germination at the growth stage "first leaf just emerging through the coleoptile" (GRO:0007059), crowns at the seedling growth stage of "first leaves unfolded" (GRO:0007060), immature inflorescences at the "third node detectable" (GRO:0007084), and embryo from the "coleoptilar stage" (PO:0001094). Total RNA was isolated from pooled tissue (10 plants) from each genotype/replication/tissue combination. The experiment was grown in a randomized complete block design with three replicates. RNA extraction, labelling, and hybridization were conducted as previously reported [52].

### GeneChip analysis

The quality of all cel files derived from the Bowman and Bowman-*cul2 *samples were ensured using GCOS v1.4 (Affymetrix, Inc. Santa Clara, CA 95051 USA). The criteria include: overall "Present" calls were more than 60%; average background values ranging from 40 to 80; the average noise values of less than 5.0 in each array; and all spike controls were called "Present" with consistent ranges among all arrays. The internal house-keeping control genes were "present", and the ratios of 3' end and 5' end of these gene expression values were under 3.0. This criterion reflected the quality of the RNA as well as the sample processing. All GeneChip data were deposited at the Plant Expression Database  with accession number BB47.

### Barley1 GeneChip datasets

Thirty-six Barley1 GeneChip data cel files were retrieved from the public Barley Natural Variation web site [47]. These are six tissue types (col, coleoptile; cro, seedling crown; gem, embryo from germinating seed; lea, seedling leaf; rad, radicle; roo, seedling root) from two genotypes, Golden Promise and Morex, with three replicates for each tissue except seedling root tissue has two replicates of Morex and four replicates of Golden Promise. We also downloaded the 10,504 SFPs discovered by Rostoks *et al*. [23,47] from this data set. In addition, twenty-one Barley1 GeneChip cel files were downloaded from Gene Expression Omnibus (GEO) database (accession GSE3170) [53], and the 2007 SFPs discovered in this data set were also retrieved from the previous report [37]. This dataset was derived from five genotypes (Barke, Morex, Steptoe, and 6 replicates of Oregon Wolf Barley (OWB) Dominant and OWB Recessive) with three replicates from whole-seedling tissue.

### Method comparison calculations

The PR curve [48] was calculated based on true positive (*TP*), false positive (*FP*), false negative (*FN*), and true positive rate (TPR) under different cutoff weight scores. Recall and TPR have the same meaning for sensitivity but different name conventions.





The comparison score was calculated using TPR, FPR, FDR, and detected SFPs. FDR is the false positives (*FP*) divided by all detected (true positives *TP *plus false positives *FP*).





Since the true positives (401) and true negatives (2200) of polymorphic probes are only inner labelled probes of total probes, the detected SFPs must be included in the score calculation.

We weighted these factors involved in sensitivity and specificity equally, and took the sum of comparison ratios of these factors. The compScore represents the percentage of improvement or drop in performance of method 1 versus method 2.



### Sequence verification

DNA fragments of Bowman and Bowman-*cul2 *were amplified from genomic DNA regions flanking the SFPs such that the amplified product for each was about 200–300 bp. The sequence information of features/probes was obtained from Affymetrix [12]. Target sequences carrying probe sites with SFPs were PCR amplified in Bowman wild type and the Bowman-*cul2 *mutant. PCR was conducted using Takara Ex Taq polymerase (Takara Shuzo Co., Kyoto, Japan) and 50 ng of DNA samples in the final volume of 50 μL following the manufacturer's protocol.

PCR was performed following this protocol: five minutes of initial denaturation at 94°C followed by 9 cycles of touch down PCR (denaturation for 30 s at 94°C, annealing starting at 63°C for 30 s and decreasing 1°C per cycle down to 55°C, and extension at 72°C for 1 min), and additional 30 cycles of PCR at 55°C annealing, 94°C for denaturation, and 72°C for extension were conducted. PCR products were electrophoresed on 1.5% agarose gel, and only single PCR products were excised from the gel for sequence validation. PCR products within excised gel pieces were purified using the Montage gel extraction kit (Millipore, Bedford, MA) following the manufacturer's protocol. Ten ng of purified DNA was sequenced using ABI PRISM^® ^3130xl Genetic Analyzer at the DNA Sequencing and Analysis Facility at the University of Minnesota.

### SFP mapping

HarvEST: barley software version 1.68 that contains Assembly #35 was downloaded from the HarvEST website [50]. The map information of SFP probe sets was obtained by directly searching Affymetrix GeneChip identifiers against Assembly #35 and blast searching against mapped EST Contig sequences. An R script was used to plot SFP probe sets on chromosomes.

## Availability and requirements

Project name: SFP discovery tool

Project home page: 

Operating system(s): Platform independent

Programming language: Java, R

Other requirements: e.g. Java 1.5.0 or higher, Tomcat 4.0 or higher

Any restrictions to use by non-academics: licence needed

## List of abbreviations

AUC: area under curve; *cul2*: *uniculm2*; FDR: false discovery rate; FPR: false positive rate; MM: mismatch; NIL: near-isogenic line; PASP: probe affinity shape power; PAOP: probe affinity outlier pursuit; PILM: probe intensity linear model; PM: perfect match; PR: precision recall; RMA: Robust Multichip Analysis; ROC: receiver operator characteristic; SAM: Significance Analysis of Microarray; SFP: single-feature polymorphism.

## Authors' contributions

WWX developed the methodology, web application, and conducted the SFP detection. SC prepared the Bowman GeneChip data and performed the PCR sequencing. SSY, YB, HB, HJ, and YX tested the SFP methodology. WWX and GJM wrote the manuscript. All authors edited the manuscript.

## Supplementary Material

Additional file 1 **Selection of affinity difference value 1**. Performances of SFP detected at different minimum values in determining the affinity difference. Three seedling crown tissue samples of Golden Promise and three seedling crown tissue samples of Morex were used in this test. Weight score cutoff 2.5 was used for all cases. Click here for file

Additional file 2 **Precision-Recall curve 2**. Precision-Recall curve using different percentiles as the base line in the weight score calculation. Click here for file

Additional file 3 **9603 SFPs 3**. 9603 Single-feature polymorphisms (SFPs) discovered from six tissue types between barley genotypes Golden Promise and Morex using the probe affinity shape power (PASP) method. Click here for file

Additional file 4 **Sensitivities and specificities 4**. The sensitivities and specificities of PASP, PAOP, and PILM methods tested at different cutoff values. Click here for file

Additional file 5 **364 SFPs 5**. 364 SFPs discovered in Bowman and Bowman-*cul2*. Click here for file

Additional file 6 **PCR primers 6**. PCR primers used for SFP validation in Bowman and Bowman-*cul2 *genomic DNAs. Click here for file

Additional file 7 **Mapping of SFPs 7**. Map information of the 91 SFP-containing probe sets in barley Bowman and Bowman-*cul2*. Click here for file
