# Supplementary material for: Single-feature polymorphism discovery by computing probe affinity shape powers
Source: BMC Genet. 2009 Aug 26;10:48. doi: 10.1186/1471-2156-10-48 (PMC2746803; doi:10.1186/1471-2156-10-48)
Supplement: Additional file 2 — Precision-Recall curve 2. Precision-Recall curve using different percentiles as the base line in the weight score calculation. [file 1471-2156-10-48-S2.pdf]

Precision-Recall curve

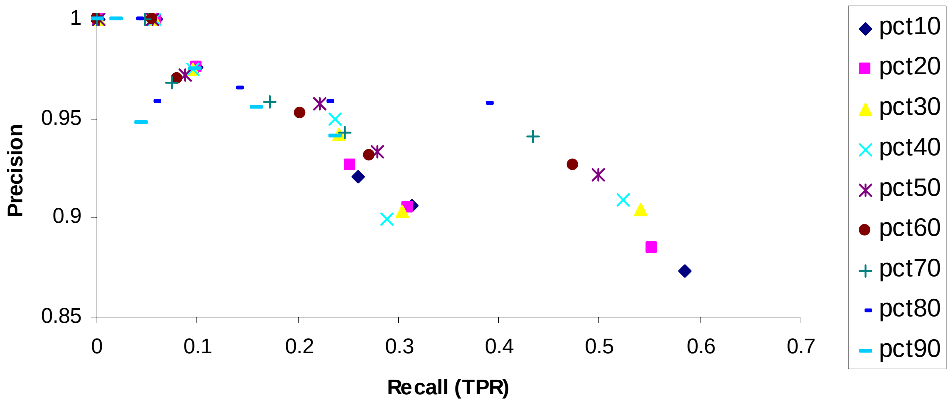

Different percentiles as base line were applied in the weight score calculation. And then the Precisions TP/ (TP+FP) and Recalls TP/ (TP+FN) at weight score cutoffs: 0, 2.5, 5.0, 10, 20, 40, and 80 for each percentile were computed. All percentiles reached the high precision (0.85 – 1.0), but the 30th at 2.5 weight score cutoff gives a 0.55 recall with a 0.9 precision at the same time. Only three seedling crown tissue samples of Golden Promise and three seedling crown tissue samples of Morex were compared in this test.
